# The complete chloroplast genome sequence of *Pennisetum centrasiaticum*, a widespread grass in Tibet, China

**DOI:** 10.1080/23802359.2021.1903355

**Published:** 2021-06-28

**Authors:** Litian Zhang, Demei Liu, Yushou Ma, Shihai Yang, Xiaoli Wang, Yanlong Wang, Ruizhen Dong

**Affiliations:** a Qinghai Academy of Animal and Veterinary Science/State Key Laboratory of Plateau Ecology and Agriculture, Qinghai University, Xining, China; b Tibet Yunwang Industrial Co., Ltd, Shigatse, China; c Shigatse Baiyacheng Agricultural Products Processing Co., Ltd, Shigatse, China; d China and Qinghai Provincial Key Laboratory of Crop Molecular Breeding Northwest Institute of Plateau Biology, Chinese Academy of Sciences, Xining, China

**Keywords:** *Pennisetum centrasiaticum*, chloroplast genome, phylogenetic analysis

## Abstract

The complete chloroplast genome of *Pennisetum centrasiaticum* was sequenced and reported here. The total genome size was 138,294 bp in length, containing a large single-copy region of 81,229 bp, a small single-copy region of 12,419 bp, and a pair of inverted repeat regions of 22,288 bp. The GC content of *P. centrasiaticum* chloroplast genome was 38.6%. It encodes a total of 119 unique genes, including 81 protein-coding genes, 34 tRNA genes, and four rRNA genes. Phylogenetic analysis showed a strong sister relationship with *Cenchrus ciliaris* and *Cenchrus purpureus*. Our findings provide fundamental information for further evolutionary and phylogenetic researches of *P. centrasiaticum*.

Chloroplast genome is exceptionally conserved in gene content and organization, providing sufficient resources for genome-wide evolutionary studies and has demonstrated the potential to resolve phylogenetic relationships at different taxonomic levels, and understand structure and functional evolution, by using the whole chloroplast genome sequences (Jansen et al. 2007; Moore et al. 2010; Ji et al. 2020).

*Pennisetum centrasiaticum* was known as an excellent pasture and had good nutritional value, widely distributed in China (Cao et al. 2010). So far, there is no report on the chloroplast genome sequence of *Pennisetum* species. Therefore, sequencing and analysis of chloroplast genome structure of *P. centrasiaticum* will contribute to a better understanding of the evolutionary mode of the chloroplast genome and provide more evidence for the identification and application of this grass. Now, we reported the complete chloroplast genome sequence of *P. centrasiaticum* based on the next-generation sequencing.

The fresh leaves of *P. centrasiaticum* were collected in Shigatse, Tibet of China (89.2506 E; 29.4411 N; masl, 4,010 m). The voucher specimen was kept in Herbarium of Shigatse Baiyacheng Agricultural Products Processing Co., Ltd. (Zhang 20200826). Genomic DNA was extracted from fresh leaves and then sequenced using the Illumina NovaSeq platform (Illumina, San Diego, CA). The raw data were used to assemble the complete cp genome using GetOrganelle software (Jin et al. 2020) with *Cenchrus ciliaris* (GenBank accession: NC041434) as the reference, and the genome annotation was performed with the program Geneious R8 (Biomatters Ltd, Auckland, New Zealand) by comparing the sequences with the cp genome of *Cenchrus ciliaris*. The theoretical sequencing coverage of the chloroplast genome is 427X. Approximately, 4.1 GB of raw data were generated with 150 bp paired-end read lengths. The complete chloroplast genome of *P. centrasiaticum* was a circular DNA molecule with 138,294 bp in length, contained a large single copy region (LSC) of 81,229 bp and a small single copy region (SSC) of 12,419 bp, as well as a pair of inverted repeat regions (IRs) of 22,288 bp. A total of 119 unique genes were annotated, including 81 protein-coding genes, 34 tRNA genes and four rRNA genes. Among them, 43 are involved in photosynthesis, and 64 genes are involved in self replication. The overall GC content of the cp genome is 38.63%, and the values in the LSC, SSC and IR regions were 36.51, 33.06, 44.06% respectively.

*Pennisetum* belonged to *Tri. Paniceae*. To confirm the phylogenetic location of *P. centrasiaticum*, complete chloroplast genome sequences of 15 species belonged to *Tri. Paniceae* were obtained from GenBank, and were aligned using MAFFT (Katoh and Standley 2013). The Neighbor-joining tree was built using MEGA7 (Kumar et al. 2016) with bootstrap set to 1,000. Phylogenetic analysis showed a strong sister relationship with *Cenchrus ciliaris* and *Cenchrus purpureus* (Figure 1). Our findings provide fundamental information for further evolutionary and phylogenetic researches of *P. centrasiaticum*.

**Figure 1.**
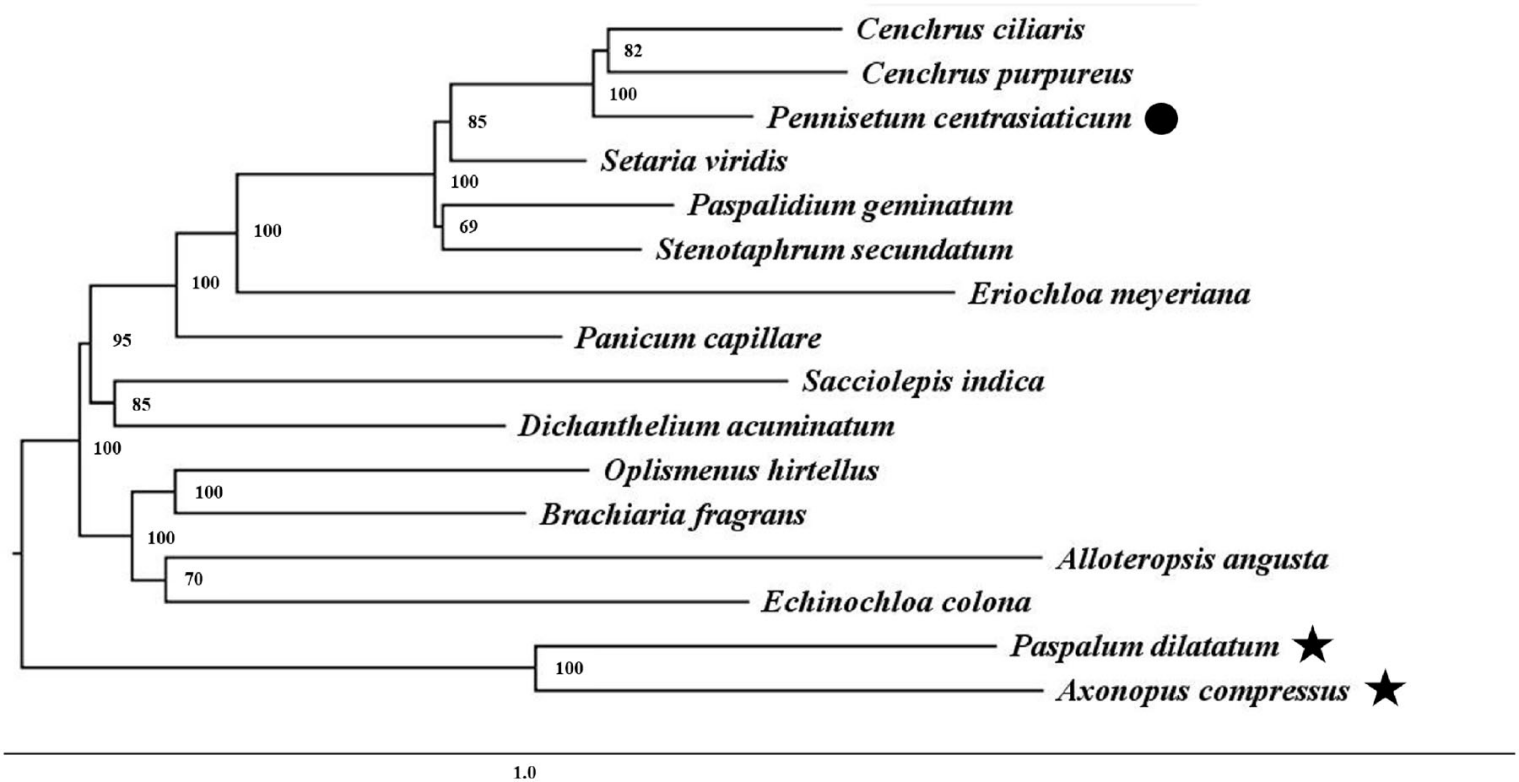
Phylogenetic relationships among 16 complete chloroplast genomes. The outgroups used to root the tree were marked with star. Bootstrap support values are given at the nodes. The analyzed species and corresponding Genbank accession numbers are as follows: *Cenchrus ciliaris*: NC041434; *Cenchrus purpureus*: NC036384; *Setaria viridis*: NC028075; *Paspalidium geminatum*: NC030494; *Stenotaphrum secundatum*: NC036704; *Eriochloa meyeriana*: NC030624; *Panicum capillare*: NC030493; *Sacciolepis indica*: NC036702; *Dichanthelium acuminatum*: NC030623; *Oplismenus hirtellus*: NC030491; *Brachiaria fragrans*: NC033879; *Alloteropsis angusta*: NC027951; *Echinochloa colona*: NC032383; *Paspalum dilatatum*: NC030614; *Axonopus compressus*: NC046490.

## Data Availability

The genome sequence data that support the findings of this study are openly available in GenBank of NCBI at (https://www.ncbi.nlm.nih.gov/) under the accession no. MW421597. The BioProject, SRA, and Bio-Sample numbers are PRJNA668466, SAMN16409664, and SRR12810250, respectively.
